# Synovitis, Acne, Pustulosis, Hyperostosis, and Osteitis Syndrome Diagnosis in Adolescent and Isotretinoin as a Possible Serious Exacerbating Factor

**DOI:** 10.7759/cureus.22776

**Published:** 2022-03-02

**Authors:** Omar O Shahada, Ahmed S Kurdi, Afnan F Aljawi, Lujain I Khayat, Anas O Shahadah

**Affiliations:** 1 Dermatology Department, King Salman bin Abdulaziz Medical City, Medina, SAU; 2 Dermatology Department, Taibah University, Medina, SAU; 3 Pediatrics Department, Prince Mohammad bin Abdulaziz Hospital, Medina, SAU

**Keywords:** hyperostosis, dermatosis, isotretinoin, acne conglobata, acne vulgaris, pustulosis, osteitis, sacroiliitis, synovitis, sapho syndrome

## Abstract

Synovitis, acne, pustulosis, hyperostosis, and osteitis (SAPHO) syndrome is a rare auto-inflammatory condition involving cutaneous and osteoarticular manifestations. This study presents a case where a 16-year-old male with glucose-6-phosphate dehydrogenase (G6PD) deficiency presented with severe nodulocystic acne after three weeks of isotretinoin therapy. In addition to worsening acne, the patient had bone and joint pain with movement restriction. The patient’s workup showed elevated erythrocyte sedimentation rate (ESR) and C-reactive protein (CRP), bilateral symmetrical sacroiliitis on magnetic resonance imaging (MRI), and multiple bony lesions on bone scintigraphy. A diagnosis of SAPHO syndrome possibly induced by isotretinoin was made. Isotretinoin discontinuation, analgesia, topical acne medications, prednisolone, and adalimumab yielded considerable clinical improvement.

## Introduction

Synovitis, acne, pustulosis, hyperostosis, and osteitis (SAPHO) syndrome is an auto-inflammatory disease characterized by both cutaneous and osteoarticular manifestations. The syndrome was first described by Chamot in 1986 and includes five conditions: synovitis, acne, pustulosis, hyperostosis, and osteitis [1]. The prevalence of this disease is <1 in 10,000 and is thought to be underestimated [2]. The disease can occur at any age, although it is more frequent between the ages 30 and 50. Females are more affected, particularly among patients under 30 years of age. SAPHO syndrome is a recurrent condition; patients experience periods of exacerbation and remission with variant severities [3]. Cutaneous and osteoarticular manifestations do not necessarily present together, and sometimes, the onset between the manifestations takes several years. Furthermore, this heterogeneous clinical presentation can lead to misdiagnosis. The usually involved sites are the axial skeleton joints and anterior chest wall. For cutaneous lesions, the most common reported lesion is palmoplantar pustulosis; other lesions include moderate-severe acne vulgaris, acne fulminans, acne conglobata, and hidradenitis suppurativa [2]. Several factors, including genetic factors, bacterial infections, and immunological events, are thought to contribute to SAPHO pathogenesis [2,4]. Isotretinoin is the first-line medication for severe acne but has been reported as an exacerbating factor for osteoarticular symptoms; however, the pathogenesis is still undefined. Isotretinoin as a provoking factor for articular symptoms in SAPHO syndrome has been rarely documented [2,5,6]. The management of SAPHO syndrome is still empirical, including NSAIDs, corticosteroids, and other immunosuppressant and immunomodulating agents. For refractory cases, the use of biological agents should be considered [7].

For further insight, we present a case of a patient with SAPHO syndrome, whose osteoarticular manifestations started after being initially treated with oral isotretinoin for severe acne.

## Case presentation

**Figure 1:**
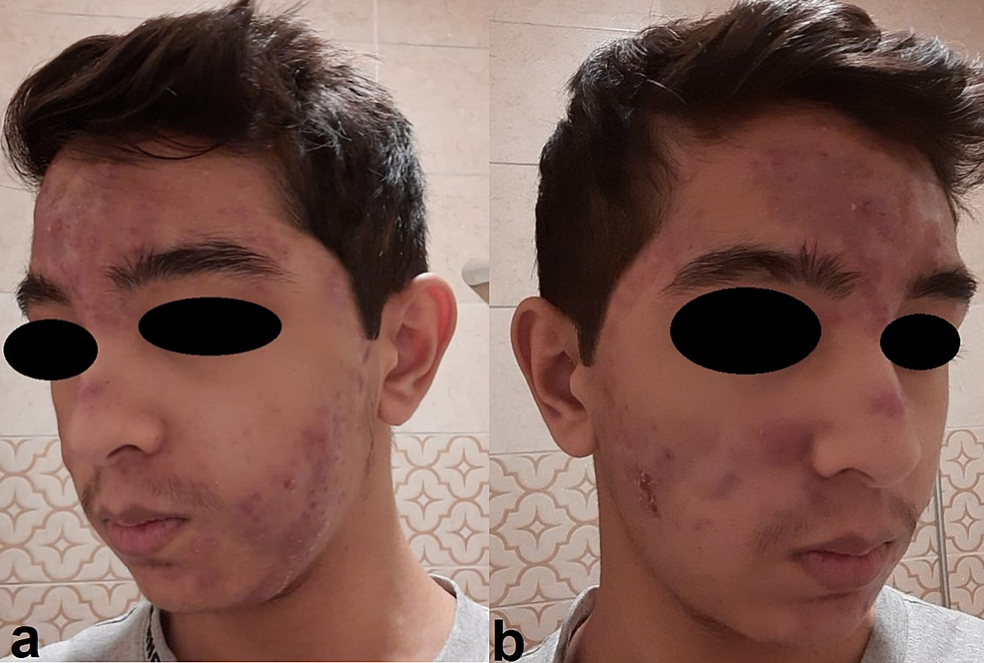
a and b: Severe nodular acne on the face.

This 16-year-old Saudi male was a known case of glucose-6-phosphate dehydrogenase (G6PD) deficiency since birth. The patient’s story started initially three months before as severe nodulocystic acne with mild joint and bone pain (Figure 1a, 1b).

The bone pain started from the hip joints up to the knee joints, with gradual onset, mild severity, and an increase with exercise and decrease with rest. The patient denied a history of fever, cough, and weight loss, and no similar presentation had been seen before. No erythema and/or warmth was observed by the patient on his joints. The dermatologist started him on isotretinoin 40 mg once daily after a normal investigation prior to isotretinoin prescription (normal complete blood count, liver enzyme, renal function test, thyroid function test, lipid profile, and hemoglobin A1C. Two weeks after starting isotretinoin, the patient started to complain of bone and joint pain with increased acne. A week later, the patient was admitted to the medical ward complaining that he could not get out of bed or move his lower limbs, with severe generalized joint and bone pain but more on his sternal and clavicle bones, lower back, and lower limps (buttocks, femur, knee, and shaft of the tibia), as well as severe acne vulgaris. The pain was progressive and severe enough to interfere with his daily work.

**Figure 2:**
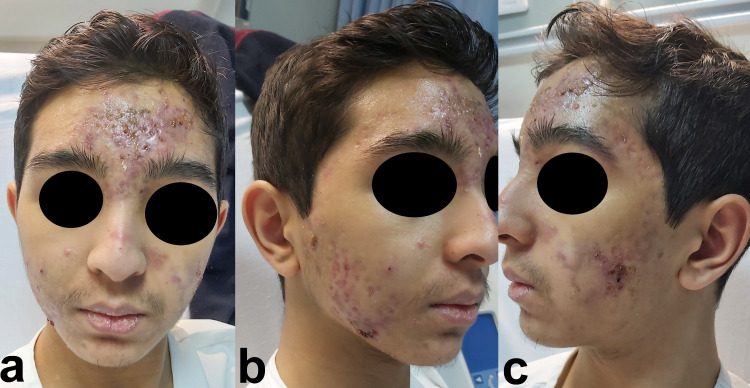
a: Acne conglobata and acne scars scatted over the face with moderate lip xerosis. b: Right side of the patient’s face with acne conglobata. c: Left side of the patient’s face with acne conglobata.

On examination, the patient was vitally stable (normal temperature, pulse rate, respiration rate, blood pressure, and oxygen saturation) and oriented to time, place, and person but looked unwell and in pain. Severe tender acne conglobata was noticed on his face, with multiple scattered acne scars and oozing of serous discharge with pustular yellow discharge from acne lesions (Figure 2a, 2b, 2c).

**Figure 3:**
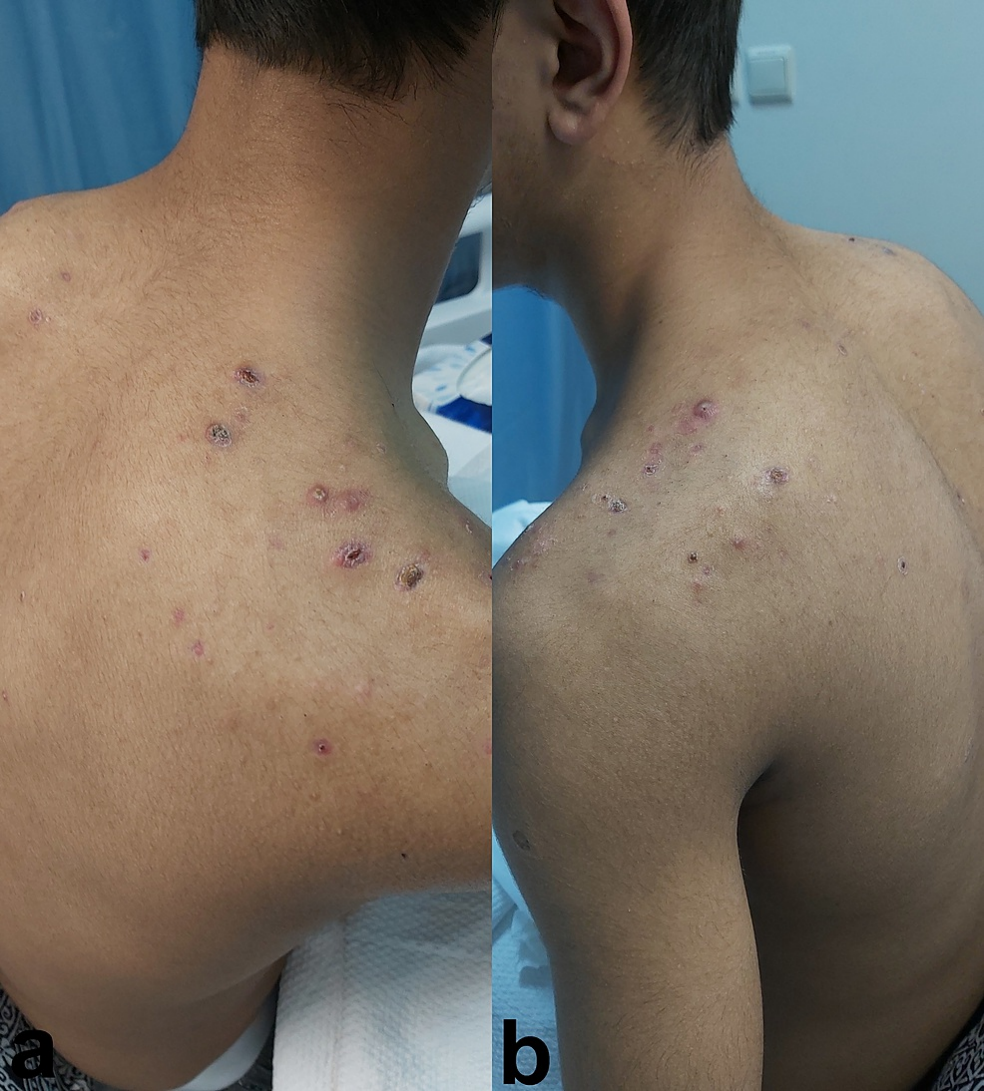
a and b: Nodular acne on the shoulders and back.

**Figure 4:**
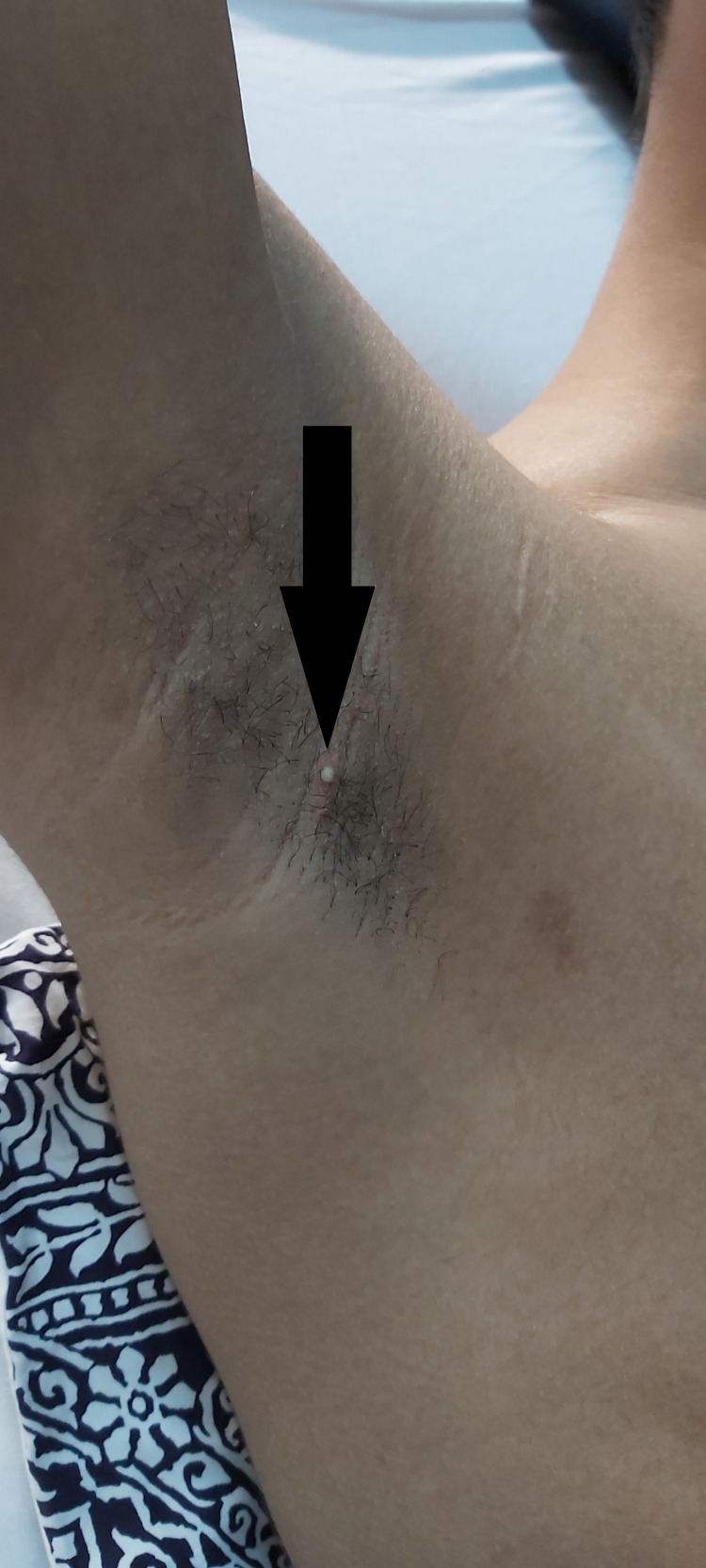
One localized pustule on the right armpit (black arrow).

Acne vulgaris was also noticed on his shoulder and back (Figure 3a, 3b) with one pustule on his right armpit (Figure 4).

**Figure 5:**
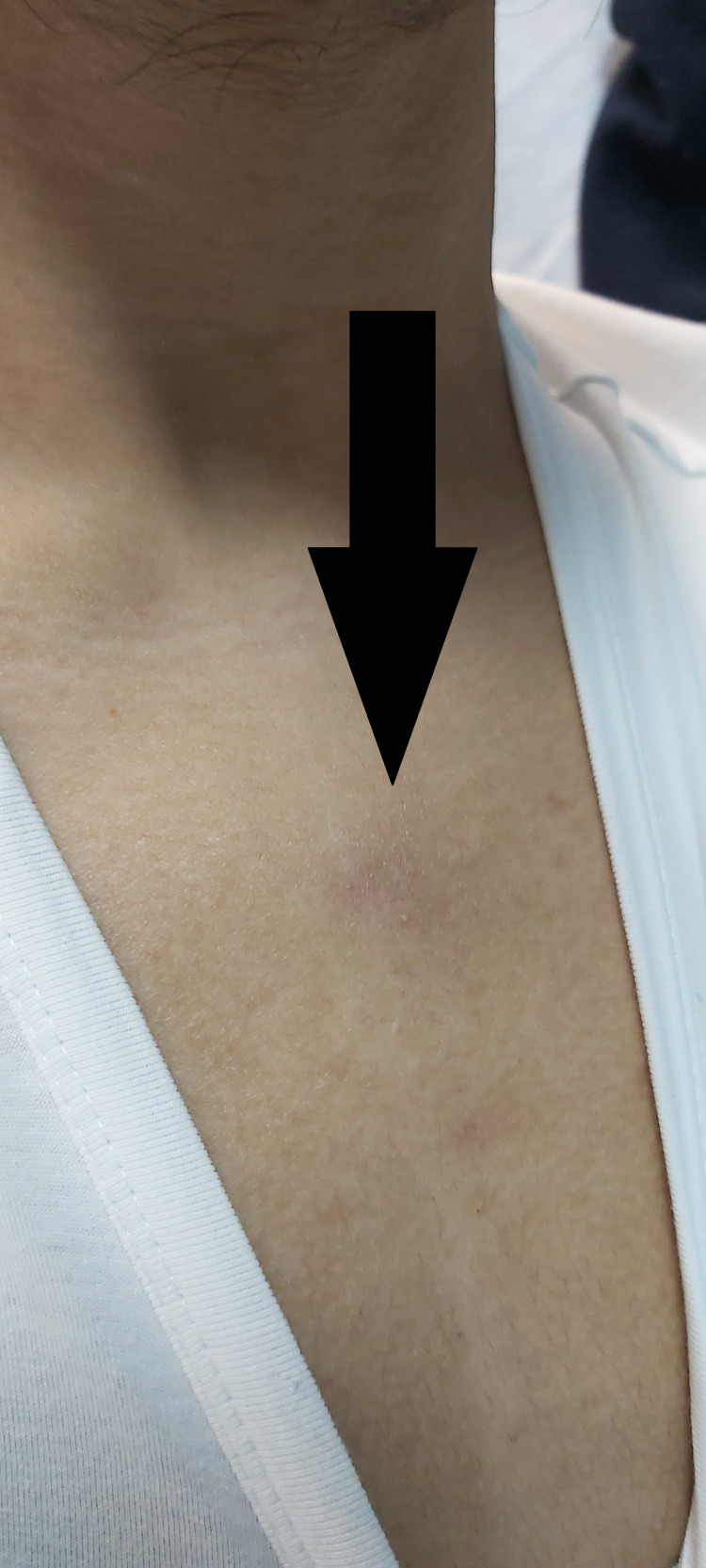
Swelling, erythema, warmth, and tenderness on the sternomanubrial joint (black arrow).

There was also tender anterior chest swelling noticed on his sternomanubrial joint (Figure 5).

The suspicion of SAPHO syndrome was raised by the dermatology team, and different blood tests and imaging were ordered.

Investigation

The complete blood count was normal, except for a slight elevation of the white blood cell (WBC) count (17.5 × 10^3^/μL; normal range: 4-11 × 10^3^/μL), erythrocyte sedimentation rate (ESR) (66 mm/hour; normal range: <30 mm/hour), C-reactive protein (CRP) (6.22 mg/dL; normal range: <0.5 mg/dL), complement component 3 (C3) (155 mg/dL; normal range: 79-152 mg/dL), and complement component 4 (C4) (45.8 mg/dL; normal range: 16-38 mg/dL).

Electrolytes, biochemistry, renal function tests, liver function tests, viral markers (HBsAg, anti-HBs AB, anti-HBe AB, HBeAg, and anti-HCV Ab), rheumatoid factor, anti-dsDNA antibody, antinuclear antibody, direct and indirect (Coombs) antiglobulin, urine culture, blood culture (primary result after 48 hours and after 5-7 days), lipid profile, and vitamin B12 (cyanocobalamin) were all normal.

**Figure 6:**
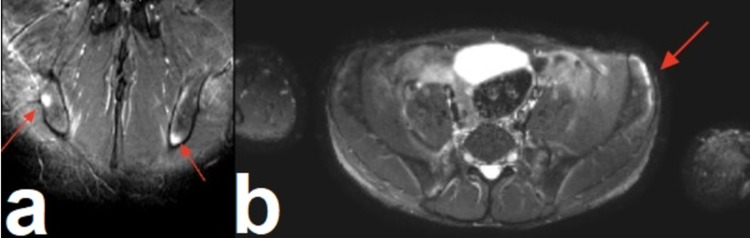
a: Bilateral osteitis at the ischio-ilial bones (red arrows). b: Osteitis of the left iliac wing (red arrow).

**Figure 7:**
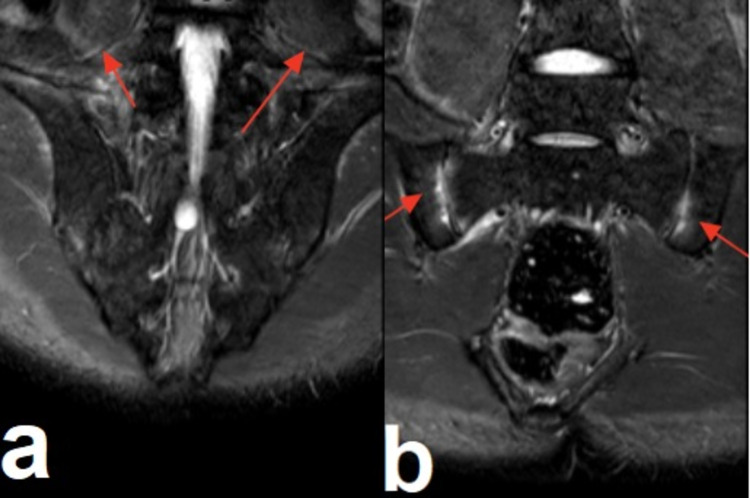
a and b: Bilateral symmetrical sacroiliitis and edema in the lumbar ligament (red arrows).

The lumbar and lower limb X-ray was normal. US of the knee joints was normal with no swelling or synovitis. Magnetic resonance imaging (MRI) showed the following, which supported the diagnosis of SAPHO syndrome: bilateral symmetrical sacroiliitis noted with mild narrowing of the joint spaces (Figure 6a, 6b) and incidentally noted small bilateral hip joint effusions, with subtle edema along the iliolumbar ligaments also seen bilaterally (Figure 7a, 7b).

**Figure 8:**
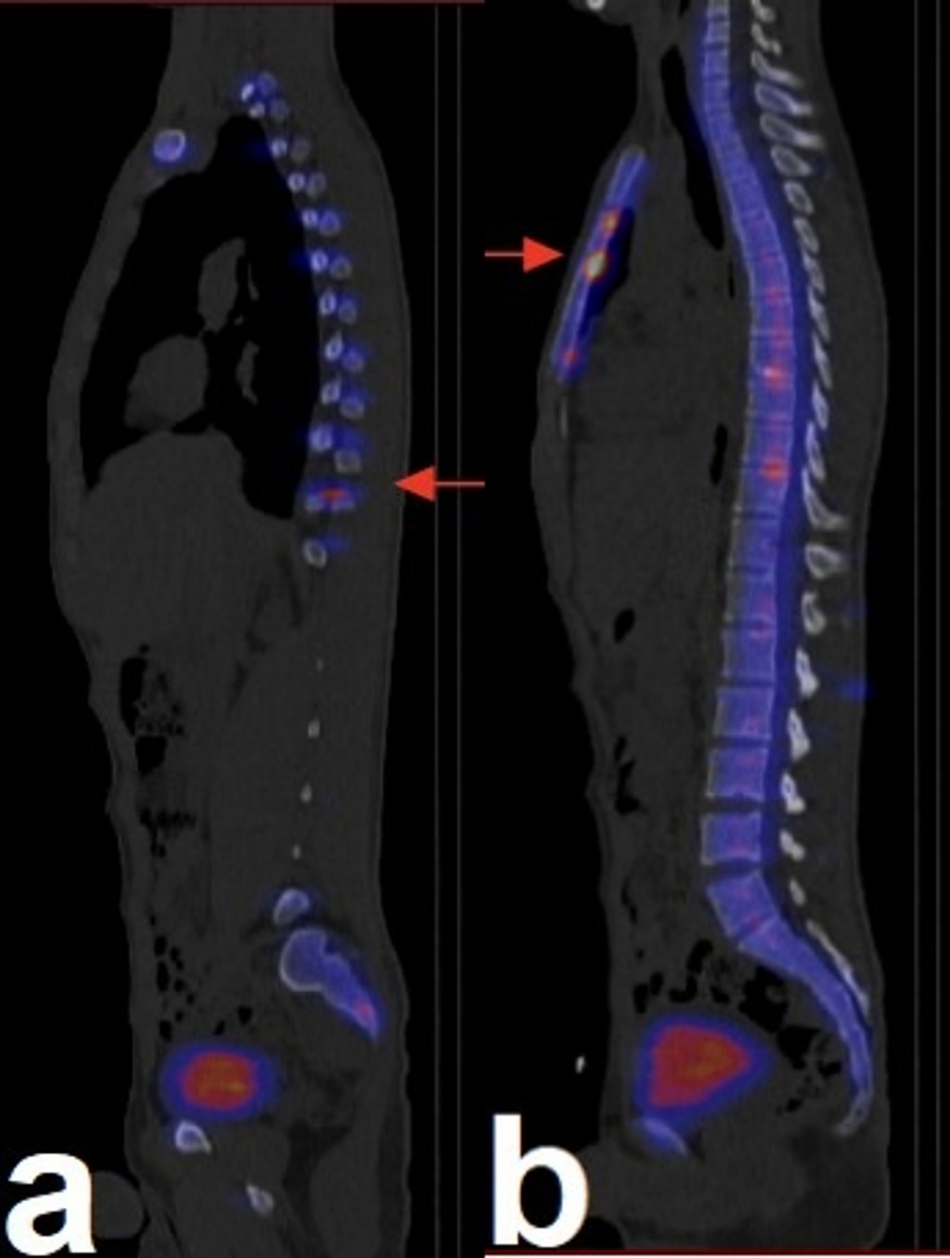
a and b: Abnormal intense radiotracer uptake in the sternum and posterior aspects of multiple ribs (red arrows).

**Figure 9:**
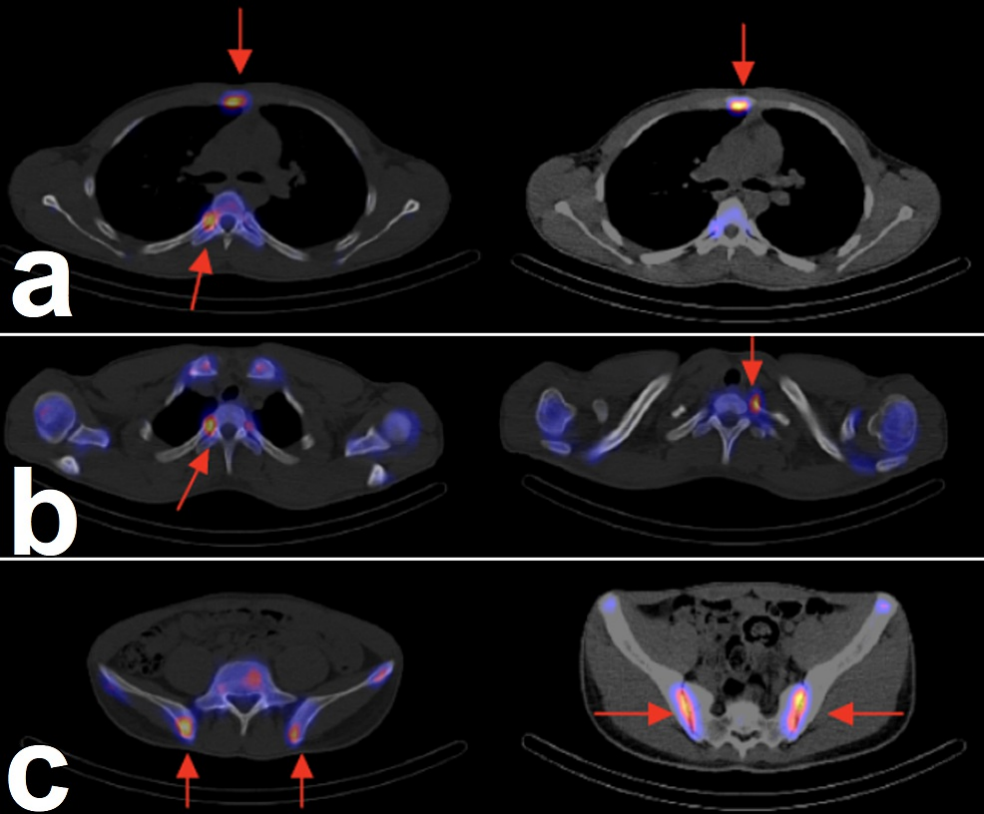
a and b: Abnormal intense radiotracer uptake in the sternum and posterior aspects of multiple ribs (red arrows). c: Abnormal intense radiotracer uptake in the sacroiliac joint (red arrows).

A three-phase bone scan, limited to both hip joints, was obtained following the IV administration of 20 mCi of technetium 99m MDP. Anterior and posterior whole-body images were then obtained at three hours. The three-phase bone scan demonstrated normal flow and blood pool images. Delayed images demonstrated homogeneous normal radiotracer uptake seen in both hip joints. The whole-body images demonstrated multiple abnormal intense radiotracer uptake in the sternum and posterior aspects involving multiple ribs (left: 1 and 5; right: 3, 6, and 11) and also both sacroiliac joints with multiple osseous lesions (Figure 8a, 8b and Figure 9a, 9b, 9c).

According to medical history, examination, and investigation, the diagnosis of SAPHO syndrome was made, and our management dermatology team decided to stop isotretinoin and prescribe oral diclofenac sodium 50 mg daily and paracetamol 1 g IV with various topical therapies including azelaic acid cream, clindamycin solution, and a combination of benzoyl peroxide with adapalene. The rheumatology team started the patient with adalimumab 40 mg injection every two weeks and oral prednisolone 15 mg once daily.

**Figure 10:**
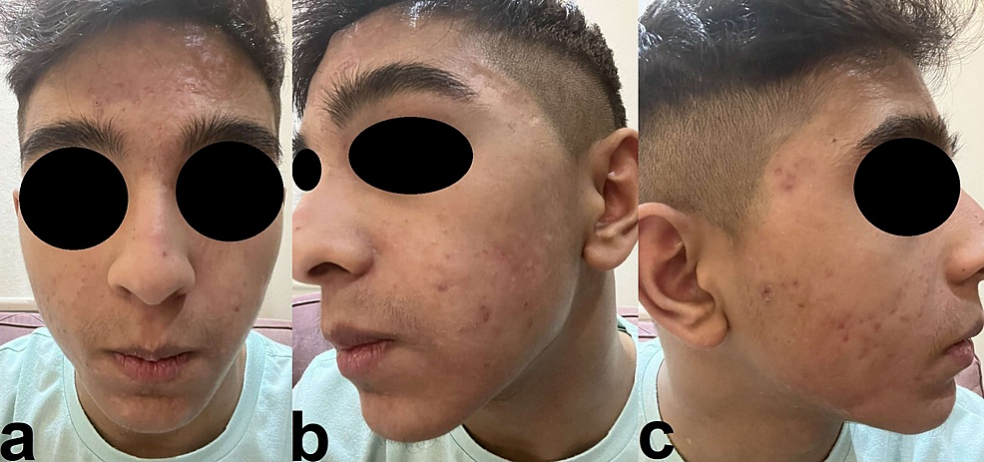
a, b, and c: No active acne lesions, acne scars, post-inflammatory hyperpigmentation, and post-acne erythema. The images were taken at the outpatient dermatology clinic.

Three months later, after eight doses of adalimumab, the patient appeared to have improved very well in both skin and musculoskeletal aspects (Figure 10a, 10b, 10c) and had a normal WBC, ESR, and CRP. The patient still needs long-term follow-up.

## Discussion

SAPHO syndrome is a rare, chronic, auto-inflammatory condition with a recurrent tendency, involving the skin, joints, and bones [1,6]. The pathogenesis of SAPHO is not well understood and could include interactions of polygenic and exogenous factors such as immune dysregulation, genetic susceptibility, and infections [2,4]. Multiple pro-inflammatory cytokines are involved, such as IL-1, IL-8, IL-18, and tumor necrosis factor (TNF)-alpha. Human leukocyte antigen (HLA) and bacterial infections have been implicated in several studies to have a possible association. The most common isolated organism from bony lesions is *Cutibacterium acnes*. The diagnosis of the syndrome is mainly clinically and is based on a combination of presenting symptoms. It is also a diagnosis of exclusion that requires laboratory investigation and the evaluation of imaging data to exclude other infectious or malignant etiologies. Neither the skin nor osteoarticular manifestations of SAPHO syndrome must coexist for a diagnosis to be made, but the symptoms usually occur within less than two years [3]. However, in some cases, there may be a time lag of several years between each manifestation [8]. Whereas osteoarticular manifestations are considered the hallmark of this syndrome and occur regardless of cutaneous involvement, osteoarticular lesions could range within osteitis, hyperostosis, synovitis, arthropathy, and enthesopathy [3]. The onset of osteoarticular involvement is usually gradual or insidious and most commonly involves the anterior chest wall (sternoclavicular and sternocostal joints) and axial skeleton joints, followed by the ankles, hips, and knees and, to a lesser extent, the small joints of the hands and feet. The distribution of affection differs with the age of onset; in children, long bones, pelvis, and lumbar spine are more affected, whereas, in adolescence, the sternoclavicular region is mainly affected [2,3]. The patient may experience pain that worsens with movement, morning stiffness, and movement restriction. Swelling, erythema, and warmth may be found in the affected sites. Arthritis is an important symptom and usually occurs in the axial joints. Fevers and fatigue are constitutional symptoms that may occur but are less common [1,6].

Cutaneous manifestations can occur in about 60% of patients and can develop with, before, or after joint involvement or may not occur at all. The most common reported manifestation is palmoplantar pustulosis, followed by severe acne, such as acne conglobata mainly among males and hidradenitis suppurativa mainly among females. Other less frequently reported cutaneous lesions include subcorneal pustular dermatosis, pyoderma gangrenosum, plaque psoriasis, and Sweet syndrome [1-3].

Isotretinoin as an exacerbating factor or a trigger for osteoarticular symptoms has been documented in some cases. The hypothesis behind this is that isotretinoin promotes joint degeneration by altering the cell membrane [9,10]. Thus, synovial cells become sensitive to minor trauma and injuries as a result of the hypersensitivity reactions isotretinoin induces in cells [11,12]. Some cases of SAPHO syndrome have been reported after isotretinoin; we gathered five different case reports, as seen in Table 1 [2,5,6,13].

**Table 1:** Comparison of five cases where isotretinoin therapy induced SAPHO syndrome.

5	4	3	2	1	
17	17	18	28	15	Age
Male	Male	Male	Male	Male	Sex
Six weeks	Four weeks	Five months	Two weeks	10 weeks	Duration of isotretinoin
Back, chest, and sternal pain	Back, chest, and hip pain	Hip pain, and sternal pain	Hip and low back pain	Low back pain	Musculoskeletal symptoms
Cystic and ulcerated acne	Ulcerated nodules on the face and trunk healing with hypertrophic scar	Nodulocystic acne in the face, chest, and back	Nodulocystic acne and abscesses in the face, neck, and back	Severe nodulocystic acne with abscesses on the face, neck, and thorax	Acne
MRI and bone scintigraphy: involvement of the sternoclavicular joint, right shoulder, sacroiliac, and long bone	MRI and bone scintigraphy: involvement of the sternoclavicular joint, sternum clavicle, L2 vertebra, left iliac crest, and left distal femoral metaphysis	X-ray and CT: widening and erosion of the sacroiliac joints, erosion of the sternum; bone scintigraphy: increased uptake in the same structures	MRI: bilateral sacroiliitis; bone scintigraphy: bull’s head sign	MRI: involvement of sternoclavicular, costoclavicular, and sacroiliac joints and spine	Imaging
Elevated ESR, CRP, and WBCs	Elevated ESR, CRP, and WBCs	Elevated ESR and CRP	Elevated CRP	Elevated ESR, CRP, and WBCs	Inflammatory markers
Prednisone pamidronate and anakinra	Prednisone pamidronate and anakinra	Isotretinoin discontinuation, restarted at a lower dose with prednisolone	Isotretinoin discontinuation and indomethacin	NSAIDs and adalimumab	Management

Furthermore, a transient exacerbation of acne may occur after the initiation of isotretinoin therapy in healthy people, but in our case, the acne was very severe, requiring the cessation of isotretinoin. The commonly reported musculoskeletal symptoms as side effects of isotretinoin therapy include back pain, arthralgia, and myalgia; other less common symptoms include calcifications, arthritis, sacroiliitis, enthesopathy, tendonitis, and hyperostosis. The majority of these side effects occur within the first six months of treatment [10-12]. However, to the extent of our knowledge, isotretinoin as an exacerbating factor or trigger in SAPHO syndrome is not commonly documented, and only a few cases have reported SAPHO syndrome induced after the introduction of isotretinoin [2,5].

Diagnosing SAPHO syndrome can be difficult due to the diverse clinical presentation. Specific validated diagnostic criteria do not exist. History, examination, and radiological and scintigraphic findings all aid in the diagnosis. Exclusion of other infectious and neoplastic conditions is required, such as osteomyelitis, bone metastases, or other spondyloarthropathies [3,4]. Imaging modalities include plain radiography, computed tomography (CT), bone scan, and magnetic resonance imaging (MRI). Plain radiographs are usually normal initially, and changes are detected in later stages. MRI detects lesions that are not visible in plain radiographs and visualizes soft tissue inflammation, so it can be used for disease activity monitoring and distinguishing between active and chronic lesions. The role of bone scintigraphy is important for surveying the entire skeleton, showing increased uptake at the involved sites, which helps locate clinically silent lesions. Uptake in sternoclavicular joints is demonstrated as a bull’s head sign that is characteristic of SAPHO syndrome. Mild anemia and nonspecific inflammatory changes can be present in laboratory investigations, such as a high erythrocyte sedimentation rate (ESR) and C-reactive protein (CRP), leucocytosis, thrombocytosis, and high complement levels [1,3,4].

There is no specific guideline for the management of SAPHO syndrome. Due to the variable clinical presentation, it depends on the individual’s presentation. Treatment is recommended for both symptom relief and prevention of complications. For patients with osteoarticular manifestations only, initial therapy with NSAIDs or oral corticosteroids for 2-4 weeks helps provide relief from pain and swelling. TNF inhibitors have also shown great benefits, such as adalimumab [1-3]. Adalimumab is also effective for severe acne vulgaris and refractory acne conglobata [14,15].

In SAPHO syndrome with moderate to severe acne, antibiotics such as oral tetracyclines may help both cutaneous and osteoarticular symptoms due to their anti-inflammatory effect [3]. For severe and scarring acne, oral isotretinoin is the first line of therapy [1,3]. However, for this patient, where isotretinoin is thought to have been an exacerbating factor for SAPHO syndrome, discontinuation is advisable [5,6,13]. Bisphosphonates, anti-IL-12/23, anti-IL-17, and anti-(IL)-1 therapy may be helpful in selected patients who are resistant to the above measures [1,3,13].

The treatment is continued depending on the course of the disease. When the patient has low disease activity, medications can be gradually reduced with close monitoring [3].

The importance of early detection and diagnosis of SAPHO syndrome is to initiate the appropriate treatment early, which is important for a favorable patient outcome and symptom relief [4].

## Conclusions

In conclusion, this case describes a 16-year-old male with severe nodulocystic acne, three weeks after isotretinoin initiation, with osteoarticular symptoms and acne vulgaris. Our case serves to remind us that the use of isotretinoin for severe acne with musculoskeletal symptoms may exacerbate both conditions and require discontinuation to manage full-blown SAPHO syndrome. Considering this study and previous ones, adolescents with acne and osteoarticular symptoms, particularly those involving the axial skeleton, should be treated with caution when considering isotretinoin initiation, and the use of an alternative, if available, should be considered.
